# Bone marrow mesenchymal stem cell-derived exosomes protect cartilage damage and relieve knee osteoarthritis pain in a rat model of osteoarthritis

**DOI:** 10.1186/s13287-020-01781-w

**Published:** 2020-07-10

**Authors:** Lei He, Tianwei He, Jianghao Xing, Qing Zhou, Lei Fan, Can Liu, Yuyong Chen, Depeng Wu, Zhenming Tian, Bin Liu, Limin Rong

**Affiliations:** 1 Department of Spine Surgery, The Third Affiliated Hospital, Sun Yat-sen University, No.600 Tianhe Road, Tianhe District, Guangzhou, 510630 Guangdong China; 2 Guangdong Provincial Center for Quality Control of Minimally Invasive Spine Surgery, No. 600 Tianhe Road, Guangzhou, 510630 China; 3 Guangdong Provincial Center for Engineering and Technology Research of Minimally Invasive Spine Surgery, No. 600 Tianhe Road, Guangzhou, 510630 China; 4 Department of Oncology, The First Affiliated Hospital, Anhui Medical University, Hefei, 230022 Anhui China; 5 Nursing Department, Lingnan Hospital, The Third Affiliated Hospital of Sun Yat-sen University, Guangzhou, 510630 China; 6 College of Materials Science and Technology, National Engineering Research Center for Tissue Restoration and Reconstruction, South China University of Technology, Guangzhou, 510630 China

**Keywords:** Osteoarthritis, Chondrocytes, BMSC-derived exosomes, Pain relief

## Abstract

**Background:**

This study aimed to investigate the effect of bone marrow mesenchymal stem cell (BMSC)-derived exosome injection on cartilage damage and pain relief in both in vitro and in vivo models of osteoarthritis (OA).

**Methods:**

The BMSCs were extracted from rat bone marrow of the femur and tibia. Chondrocytes were treated with IL-1β to establish the in vitro model of OA. Chondrocyte proliferation and migration were assessed by CCK-8 and transwell assay, respectively. A rat model of OA was established by injection of sodium iodoacetate. At 6 weeks after the model was established, the knee joint specimens and dorsal root ganglion (DRG) of rats were collected for histologic analyses. For pain assessment, paw withdrawal threshold (PWT) and paw withdrawal latency (PWL) were evaluated before model establishment and at 1, 2, 4, and 6 weeks after model establishment.

**Results:**

Exosomes can be endocytosed with the chondrocytes in vitro. Exosome treatment significantly attenuated the inhibitory effect of IL-1β on the proliferation and migration of chondrocytes. Exosome pre-treatment significantly attenuated IL-1β-induced downregulation of COL2A1 and ACAN and upregulation of MMP13 and ADAMTS5. In the animal study, exosome treatment significantly upregulated COL2A1 protein and downregulated MMP13 protein in the cartilage tissue of the OA rat. At weeks 2, 4, and 6, the PWL value was significantly improved in the exosome-treated OA rats as compared with the untreated OA animals. Moreover, exosome treatment significantly alleviated the upregulation of CGRP and iNOS in the DRG tissue of OA rats.

**Conclusion:**

BMSC-derived exosomes can effectively promote cartilage repair and extracellular matrix synthesis, as well as alleviate knee pain in the OA rats.

## Background

Osteoarthritis (OA) is the most common degenerative joint disorder, characterized by progressive articular cartilage degradation, subchondral bone thickening, osteophyte formation, synovial inflammation, and calcification of ligaments [1]. OA has a multifactorial etiology, including joint injury, aging, obesity, and heredity [2–5]. The molecular mechanism of OA pathogenesis remains poorly understood, but both chondrocytes [6] and inflammation [7] are considered to play important roles. Current treatment options for OA include antalgics and non-steroid anti-inflammatory drugs [8]. However, these treatments are symptomatic and non-curative, only aiming for pain reduction and symptom control [9]. Surgical interventions, such as total knee or hip arthroplasty, could relieve pain and deformity, but also induce postoperative complications [10, 11].

Mesenchymal stem cells (MSCs) possess multilineage differentiation potential and have been applied in cell therapy for OA. Animal studies have revealed the therapeutic efficacy of MSC intraarticular injections for animal models of OA [12–14]. The regenerative effects of MSCs are attributed to its paracrine mechanisms with anti-inflammatory and chondroprotective effects [15, 16]. Nevertheless, accumulating evidence has suggested that many of the regenerative properties previously credited to MSCs should be attributed to the secreted exosomes [17–19]. MSCs produce massive amounts of exosomes which are membrane vesicles playing an important role in cell-to-cell communication [20, 21]. In addition, MSC-derived exosome transplantation possesses several advantages, such as non-immunogenicity, non-tumorigenicity, and convenient storage and transportation, as compared with MSC therapy [22, 23]. Therefore, MSC-derived exosomes may be a good alternative for MSC therapy.

Although considerable advances have been demonstrated in several disease models, MSC-derived exosomes (EXO) have just been applied in OA therapy within recent 3 years [24–28]. In addition, the effect of exosomes on pain relief in OA animals is still unknown. We hypothesized injection of bone marrow mesenchymal stem cell (BMSC)-derived exosomes into the knee joint capsule may relieve knee OA pain and promote cartilage repair and extracellular matrix synthesis. Therefore, this study aimed to investigate the effect of BMSC-derived exosomes on cartilage damage and pain relief using both in vitro and in vivo models of osteoarthritis (OA).

## Methods

### Ethics statement

All experimental procedures and animal handling were performed with the approval of the Animal Care and Use Committee of Sun Yat-sen University, in accordance with the National Institutes of Health Guide for the Care and Use of Laboratory Animals.

### Isolation and characterization of rat BMSCs

Rat BMSCs were isolated from 2-week-old Sprague-Dawley rats’ femurs (*n* = 2, provided by the Guangdong Medical Laboratory Animal Center) according to the previous literature [29]. Dulbecco’s modified Eagle’s medium(DMEM) supplemented with 10% fetal bovine serum(FBS) (Gibco, USA) containing bone marrow cells was cultured at 37 °C, 5% CO_2_ cell culture incubator. The medium was replaced for the first time after 24–48 h and was replaced every 3 days afterward. Passage 3–5 (P3–P5) cells were used for subsequent experiments.

For phenotype characterization, BMSCs were stained with rabbit anti-rat polyclonal antibodies CD29, CD44, CD90, CD11, and CD45 (eBioscience Inc., USA) and analyzed by flow cytometry (BD FACSCanto™, USA).

For determining the multipotential differentiation capabilities of rat BMSCs, including osteogenic, adipogenic, and chondrogenic differentiation, BMSCs were cultured in the following medium types: (1) osteogenic differentiation medium—high-glucose DMEM, 10% FBS, 50 μg/ml ascorbic acid, 10 mM β-glycerophosphate, 10 nM dexamethasone, 100 U/ml streptomycin, and 100 U/ml penicillin(Sigma-Aldrich, USA); (2) adipogenic differentiation medium—high-glucose DMEM, 10% FBS, 0.1 mmol/l 3-isobutyl-1-methylxanthine, 10 μg/ml insulin, 10 nM dexamethasone, 50 μg/ml indomethacin, 100 U/ml streptomycin, and 100 U/ml penicillin(Sigma-Aldrich, USA); and (3) chondrogenic differentiation medium—high-glucose DMEM, 50 μg/ml ascorbic acid, 100 nM dexamethasone, 1 mM sodium pyruvate, 40 μg/ml proline, 100 U/ml streptomycin, 100 U/ml penicillin (Sigma-Aldrich, USA), 10 ng/ml TGFβ3 (PeproTech, USA), ITS + premix (final concentrations, 6.25 μg/ml bovine insulin, 6.25 μg/ml transferrin, 6.25 μg/ml selenous acid, 5.33 μg/ml linoleic acid, and 1.25 mg/ml bovine serum albumin) (BD biosciences, USA). The induction medium was changed every 3 days. At day 14, cells were fixed and stained with Alizarin Red S for osteocytes, Oil Red O for adipocytes, and Alcian Blue for pellet culture chondrocytes (Leagene Biotechnology, China).

### Isolation and characterization of BMSC-derived exosomes

Rat BMSCs were cultured in low-glucose DMEM containing 10% exosome-free FBS (Gibco, USA) for 48 h to collect conditioned medium. The supernatant was then centrifuged at 110,000*g* at 4 °C for 1 h using a 45 Ti rotor (Beckman Coulter, USA). The resulting pellets were washed and resuspended in PBS, followed by centrifugation at 110,000*g* at 4 °C for 1 h.

The exosome morphology was observed under 100-kV transmission electron microscopy (TEM, HITACHI H-7000FA, Japan). The particle size distribution of exosomes was analyzed by Zetasizer Nano (Malvern, UK). Antibodies against CD63 (ProteinTech, USA), TSG101 (Abcam, UK), and Flotillin-1 (Abcam, UK) were used to identify the protein-level expressions by western blot.

### Primary culture of chondrocytes and in vitro model of OA-like chondrocytes

Rat chondrocytes were isolated from 1-week-old Sprague-Dawley rats’ ribs (*n* = 2, provided by the Guangdong Medical Laboratory Animal Center) according to the previous literature [30]. The resultant cells were cultured with DMEM/F-12 medium containing 10% FBS, 100 U/ml penicillin, and 100 U/ml streptomycin (Gibco, USA). The medium was changed every 3 days. For all experiments described, the chondrocytes in monolayer culture were used between passages 2–3.

For the in vitro model of OA-like chondrocytes, chondrocytes were induced to express an OA-like phenotype by IL-1β treatment (Peprotech, USA). Briefly, IL-1β (10 ng/ml) was added to the chondrocytes medium for 24 h.

### BMSC-derived exosome uptake in vitro and in vivo

Exosomes were labeled using the red fluorescent dye PKH26 according to the manufacturer’s instructions (Sigma-Aldrich, USA). Excess dye from the labeled exosomes was removed by ultracentrifugation at 100,000*g* for 1 h at 4 °C using a 32 Ti rotor (Beckman Coulter, USA), and the exosome pellets were washed three times by PBS. The final pellets were resuspended in PBS. Exosomes were co-cultured with rat chondrocytes at a concentration of 10 μg/ml in serum-free medium at 37 °C for 12 h and then fixed with 4% paraformaldehyde. The nuclei were stained with Hoechst 33342 (10 μg/ml, Beyotime, China). The cytoskeleton was stained by Actin-Tracker Green (Beyotime, China). The uptake of exosome was observed using a confocal laser scanning microscope (Zeiss LSM710, Germany).

For the evaluation of exosome uptake in vivo, labeled exosomes (40 μg/100 μl) were injected into the joint cavity after the rat model of OA was established. Small animal fluorescence imager (eXplore Optix, Advanced Research Technology, USA) was used to monitor the signals in exosomes.

### Real-time RT-PCR

Total RNA was extracted from cells using the Total RNA Kit I (Omega Bio-Tek, USA), followed by reversely transcribed to generate the first-strand cDNA using the PrimeScript RT reagent Kit (Takara, Japan) according to the manufacturer’s protocol. Quantitative PCR was performed using the SYBR Green PCR mix (Takara, Japan) on a Bio-Rad CFX Connect real-time system (Bio-Rad, USA). The primers were as follows: MMP13 (forward 5′-AGCAGGTTGAGCCTGAACTGT-3′and reverse 5′-GCAGCACTGAGCCTTTTCACC-3′), ADAMTS5 (forward 5′-ACGCGGGACCTCAGACGTGGTG-3′and reverse 5′-TCGTGGCCGCGTTCTTGCTCAC-3′), COL2A1 (forward 5′-GCCCAACTGGCAAACAAGGAGAC-3′ and reverse 5′-GCAGGGCCAGAAGTACCCTGATC-3′), COL1A1 (forward 5′-CCGTGACCTCAAGATGTGCC-3′ and reverse 5′-GAACCTTCGCTTCCATACTCG-3′), ACAN (forward 5′-GGCTTCCCACCGTCCCAGCAG-3′ and reverse 5′-GAAGTGTCTGTGCTGCCTGTGAA-3′), TGFβ1 (forward 5′-GTGGCTGAACCAAGGAGACG-3′ and reverse 5′-AGGTGTTGAGCCCTTTCCAG-3)′, PCNA (forward 5′-GGGCTGAAGATAATGCTGATACC-3′ and reverse 5′-ATGTTCCCATTGCCAAGCTC-3′), Casp3 (forward 5′-GTATGCTTACTCTACCGCACCC-3′ and reverse 5′-CAGGGAGAAGGACTCAAATTCC-3′), and GAPDH (forward 5′-CCTGGAGAAACCTGCCAAGTAT-3′ and reverse 5′-TAGCCCAGGATGCCCTTTAGT-3′). GAPDH was regarded as a reference gene. The PCR reaction experiment of each sample was repeated three times, and the RT-PCR data was analyzed by the 2^−ΔΔCt^ method.

### Western blot

Passage 2–3 chondrocytes were used for protein extraction. The method for collagen type II (COL2A1) extraction was as described previously [27]. Briefly, chondrocytes were washed with PBS three times and lysed with RIPA lysis buffer (Beyotime, China) supplemented with 1 mM protease inhibitor cocktail and 1 mM phosphatase inhibitor cocktail (Thermo, USA). The mixture was homogenized and lysed on ice and centrifuged at 12000 r/min for 30 min, 4 °C. The resulting supernatant was collected. Protein concentration was determined by the BCA method. The same amount of protein sample was electrophoresed and transferred to the polyvinylidene fluoride (PVDF) membrane (Millipore, USA). The membrane was blocked with 5% skim milk for 1 h at room temperature, and incubated at 4 °C overnight with the primary antibody against COL2A1 (Abcam, UK), MMP-13 (Abcam, UK), CGRP (GeneTex, USA), iNOS (GeneTex, USA), COL1A1 (Abcam, UK), TGFβ1 (Abcam, UK), PCNA (Abcam, UK), Casp3 (Abcam, UK), and GAPDH (Abclonal, USA). After washing, the membrane was incubated with the secondary antibodies (Abcam, UK) at room temperature for 1 h. Integrated density for protein bands was determined using Tanon 5200 (Tanon Science &Technology, China).

### Immunofluorescence

Rat chondrocytes were fixed in 4% paraformaldehyde at room temperature for 30 min, followed by 0.5% Triton X-100/PBS for 30 min at room temperature. The cells were blocked with 5% BSA for 1 h at room temperature. The cells were incubated with primary antibodies COL2A1 (Abcam, UK) and MMP13 (Abcam, UK) overnight at 4 °C. After extensive washing, the cells were incubated with secondary antibodies including Alexa Fluor 488 (Thermo Fisher, USA). The nucleus was stained with Hoechst 33342 (10 μg/ml, Beyotime, China) for 10 min at room temperature. The cells were observed using a laser confocal microscope (Zeiss LSM710, Germany).

### Cell proliferation and migration assay

Chondrocytes were seeded in a 96-well plate at a concentration of 100 μl/well (1 × 10^4^ cells), and cultured in a 37 °C for 24 h. The 96-well plates were incubated at 37 °C for 24 h or 48 h. CCK-8 solution of 10 μl (CCK-8, Dojindo, Japan) was added to each well, and the cells were incubated at 37 °C for 4 h, followed by detecting the optical density at a wavelength of 450 nm by an enzyme-labeling instrument (SpectraMax M5, USA).

The migration ability of cells was determined by the transwell invasion assay. Chondrocytes (1 × 10^6^ cells, in 100 μl serum-free medium) were added to the upper chamber in a 24-well plate, in which the lower chamber contained 600 μl of complete medium. After 12 h, the invaded cells were fixed with 4% paraformaldehyde for 15 min, followed by staining with 1% crystal violet staining solution (Sangon Biotech, China). The cells were photographed by a fluorescence microscope (Olympus IX71, Japan).

### The rat model of OA and experimental design

Twenty-four 10-week-old male SD rats (provided by the Guangdong Medical Laboratory Animal Center) were anesthetized with 2.5–3% isoflurane. Sixteen rats were performed the unilateral intraarticular injection with 8% sodium iodoacetate (2 mg in 50 μl saline, Sangon Biotech, China) to produce the OA change of the knee, and the rats in the sham group were performed with joint puncture only. One week later, intraarticular injection of BMSC-derived exosomes (40 μg/100 μl) was performed in the OA rats once a week (*n* = 8, OA + EXO group), while intraarticular injection of 100 μl normal saline was performed in the sham group (*n* = 8) and OA group (*n* = 8). At 6 weeks after surgery, the knee joint specimens and dorsal root ganglion (DRG) of rats were collected for histologic analysis and western blot. All the in vivo data for cartilage repair and pain relief were done with the same set of animals.

### Histological staining and immunohistochemistry

At 6 weeks after treatment, the rats were sacrificed and articular cartilage samples were collected. After fixation with paraformaldehyde for 24 h and decalcified for 21 days in 10% EDTA (pH 7.4), tissues were embedded in paraffin and sectioned into a 5-μm-thick section. The serial sections were obtained from the medial and lateral compartments at 200-μm intervals. The selected sections were deparaffinized in xylene, rehydrated through a graded series of ethanol washes, and followed by hematoxylin and eosin (H&E) and Safranin O/Fast Green staining (Servicebio, China). The degree of cartilage degeneration was assessed on the medial and lateral tibial plateau joint with the Osteoarthritis Research Society International (OARSI) score [31].

For immunohistochemistry (IHC), the deparaffinized sections were soaked in EDTA (pH 9.0) for antigen retrieval by a microwave method. The sections were placed in a 3% hydrogen peroxide solution and incubated at room temperature for 25 min in the dark, followed by blocking with 3% BSA at room temperature for 30 min. Then, the sections were incubated with primary antibody COL2A1 (Abcam, UK), MMP13 (Abcam, UK), and COL1A1 (Abcam, UK) at 4 °C overnight, followed by the secondary antibody(Abcam, UK) at room temperature for 60 min the next day. After extensive washing, 3,3′-diaminobenzidine (DAB)-peroxidase substrate and hematoxylin solution (Servicebio, China) was added.

### Pain assessment

The mechanical paw withdrawal threshold (PWT) and the thermal paw withdrawal latency (PWL) of rat models were measured by Von-Frey filaments and thermal radiometer to evaluate mechanical and thermal allodynia [32, 33]. The baseline PWT and PWL were measured before the OA model establishment and at 1, 2, 4, and 6 weeks after model establishment. Antibodies against CGRP (GeneTex, USA) and iNOS (GeneTex, USA) were used to identify the protein level expressions of DRG tissues by immunofluorescence and western blot.

For PWT measurement, the von Frey fibers (Aesthesio, Italy) were used to vertically stimulate the center of the rat hind paw with increasing intensity. The rat quickly flinched or licked the paw indicated a positive withdrawal reaction, and then the adjacent decreasing intensity was selected to give a stimulation. If the withdrawal reaction was negative, the adjacent fibril with increasing stimulation intensity was used to give a stimulation. Until the withdrawal reaction was positive, and there are 3 positive withdrawal reactions within the 5 consecutive stimuli, the von Frey fibers value was defined as PWT.

For PWL measurement, the hind paw of the rat was stimulated with an automatic Plantar Test (Hargreaves Apparatus, Italy) and its thermal threshold was measured. The rat was placed on clear glass, covered with a transparent cover, and the infrared heat source was applied to the surface of the hind paw. PWL was defined as the time interval between the start of thermal stimulation and the paw withdrawal. Each rat was recorded 3 times with an interval of at least 5 min between the two adjacent measurements.

For immunofluorescence and western blot, the rats were sacrificed after 6 weeks of treatment. Lumbar DRGs at levels L3–L5 were dissected from the surrounding tissue, and cardiac perfusion was performed with PBS followed by 4% paraformaldehyde. The DRG tissues were collected for frozen sections or protein extractions. The immunofluorescence or western blot procedure and antibodies used to analyze were described before. The fluorescent secondary antibody including Alexa Fluor 488 and 594 (Thermo Fisher, USA) were used for immunofluorescence of frozen sections.

### Statistical analysis

Data are expressed as mean ± standard deviation (SD). Repeated measures were analyzed by repeated measured analysis of variance (ANOVA) with Bonferroni post hoc analysis. The other data were analyzed by one-way ANOVA with Bonferroni or LSD post hoc analysis. All statistical analyses were performed using the IBM SPSS software (SPSS Statistics V22, IBM Corporation, USA). *P* values < 0.05 were considered statistically significant.

## Results

### Characterization of BMSCs and BMSC-derived exosomes

The BMSCs were extracted from the rat bone marrow. Flow cytometry analysis showed that BMSCs were positive for mesenchymal markers, including CD29, CD44, and CD90, but negative for CD11b and CD45 (Supplementary Fig. 1A). In addition, the multilineage differentiation potential of BMSCs was demonstrated by Alizarin Red staining, Oil Red O staining, Alcian Blue staining, and Safranin O staining (Supplementary Fig. 1B).

For exosome preparation, 200 ml of BMSCs conditioned medium was centrifuged, and 100–150 μg of exosomes can be purified. The dynamic light-scattering measurement indicated that the mean size of BMSC-derived exosomes was 153 nm (Supplementary Fig. 1C). TEM showed that the exosomes exhibited an ova shape (Supplementary Fig. 1D). For western blot of exosomal surface markers, 20 μg of BMSCs or exosomes was loaded onto SDS-PAGE. Western blot analysis indicated that these vesicles displayed exosomal surface markers, including Flotillin-1, TSG101, and CD63, and were negative for the non-exosomal marker (Calnexin) (Supplementary Fig. 1E).

### Exosomes attenuated IL-1β-induced inhibitions on the proliferation and migration of chondrocytes

**Fig. 1:**
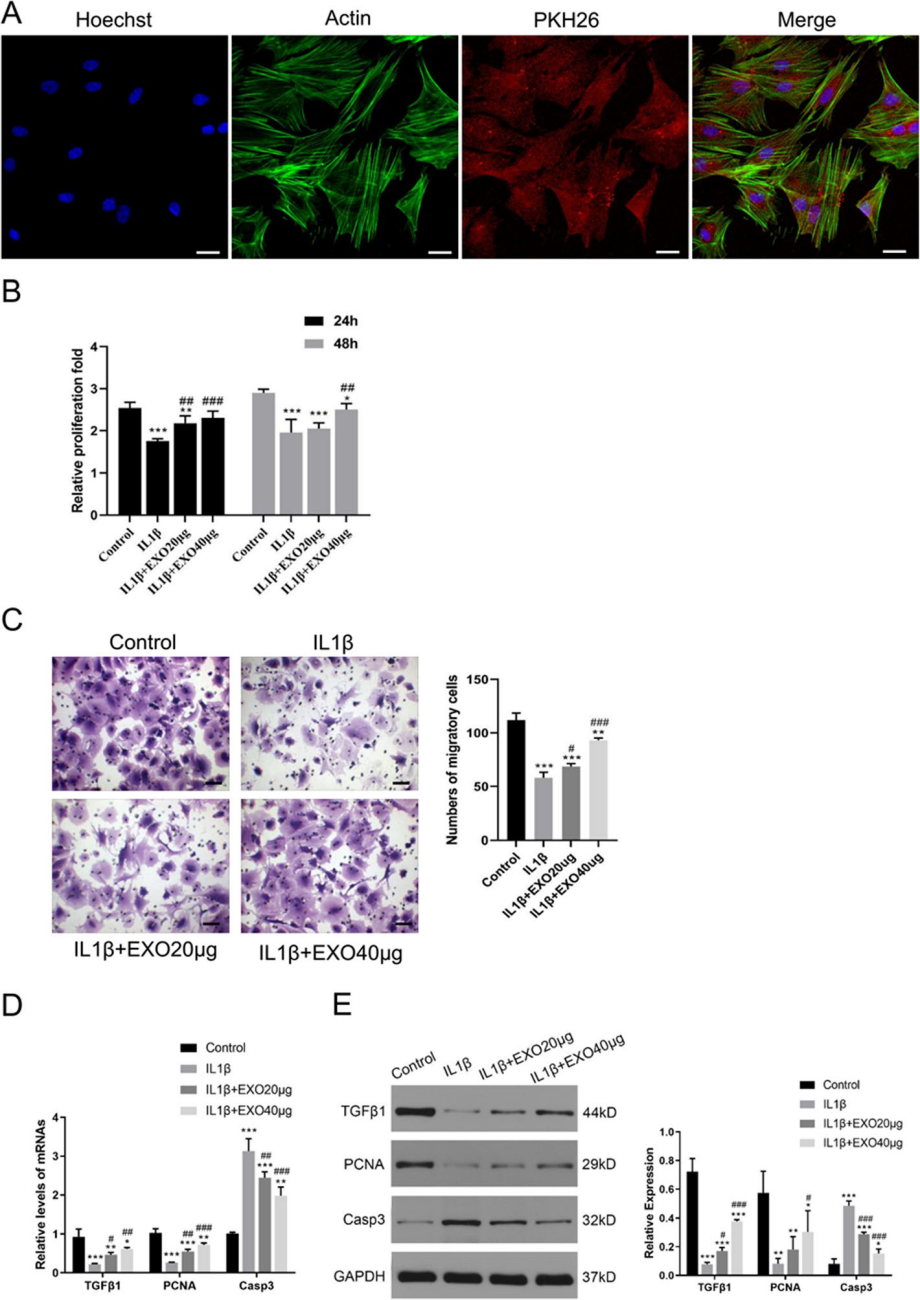
Exosomes attenuated IL-1β-induced inhibitions on the proliferation and migration of chondrocytes. **a** Immunofluorescence staining of chondrocytes and BMSC-derived exosomes (PKH26) showed that exosomes gathered inside the chondrocytes. **b** The proliferation of chondrocytes was evaluated by CCK-8 assay. **c** Chondrocyte migration was determined by the transwell migration assay. Chondrocytes (1 × 10^6^ cells, in 100 μl serum-free medium) were added to the upper chamber in a 24-well plate, in which the lower chamber contained 600 μl of complete medium. Five fields were randomly selected from each sample for quantification. All experiments were repeated independently at least three biological replicates. In the in vitro model of chondrocyte degeneration, PCR (**d**) and western blot (**e**) assays were performed to determine the mRNA and protein levels of growth factor (TGFβ1), proliferation marker (PCNA), and apoptosis marker (Casp3). Scale bar = 50 μm. *< 0.05, **< 0.01, ***< 0.001, compared with the control group. ^#^< 0.05, ^##^< 0.01, ^###^< 0.001, compared with the IL-1β group

Next, the effect of exosomes on chondrocytes was evaluated. Confocal microscopy images showed that when co-cultured with chondrocytes, BMSC-derived exosomes gathered inside the chondrocytes, and fluorescence was observed in the whole chondrocytes (Fig. 1a), suggesting that some exosomes were endocytosed into the chondrocytes. In addition, the endocytosis of exosomes by chondrocytes was measured at different time points (6, 12, 24 h). With the increase of time, the amount of exosome endocytosis by chondrocytes gradually increased (Supplementary Fig. 2A, 2B). We also performed PKH26 staining on BMSCs and compared them with BMSCs with exosomal endocytosis to further demonstrate the morphological characteristic of exosomal components in chondrocytes (Supplementary Fig. 2C).

Proinflammatory cytokine IL-1β is one of the critical mediators of cartilage destruction in OA [34]. CCK-8 assay showed that inflammatory cytokine IL-1β treatment significantly reduced the proliferation of chondrocytes (*P* < 0.001, Fig. 1b). However, at 24 or 48 h, BMSC-derived exosomes, especially 40 μg exosomes, significantly attenuated IL-1β-induced inhibition on chondrocyte proliferation (*P* < 0.01, Fig. 1b).

Likewise, transwell migration assay also demonstrated that IL-1β treatment significantly reduced the migration of chondrocytes (*P* < 0.001, Fig. 1c), whereas BMSC-derived exosomes (20 μg and 40 μg) significantly attenuated IL-β-induced inhibition on the migration of chondrocytes (*P* < 0.05, Fig. 1c).

In the in vitro model of chondrocyte degeneration, PCR (Fig. 1d) and western blot (Fig. 1e) assays demonstrated that exosome treatment can attenuate IL-1β-induced downregulation of growth factor (TGFβ1) and proliferation marker (PCNA) expression, as well as upregulation of apoptosis marker (Casp3) (all *P* < 0.05).

### Exosomes attenuated IL-1β-induced downregulation of anabolic markers and upregulation of catabolic markers in cartilage degradation

**Fig. 2:**
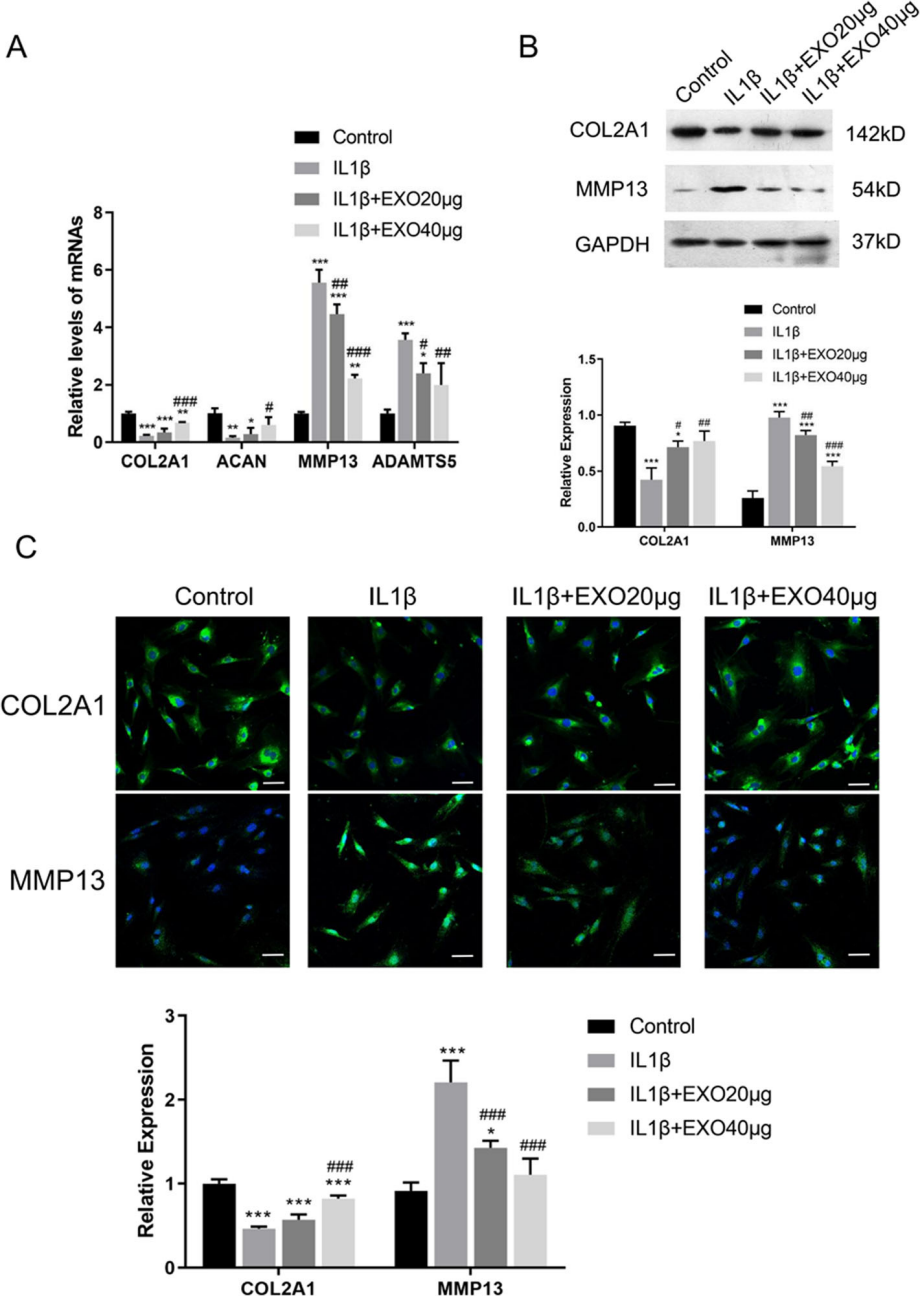
Exosomes attenuated IL-1β-induced downregulation of anabolic markers and upregulation of catabolic markers in cartilage degradation. **a** mRNA levels of MMP13, ADAMTS5, COL2A1, and ACAN were determined by RT-PCR. **b** Western blot analysis of protein levels of COL2A1 and MMP13. **c** Immunofluorescence staining of COL2A1 and MMP13. Rat chondrocytes were seeded at a density of 1 × 10^4^ cells/well in the glass bottom culture dishes. Five fields were randomly selected from each sample for quantification. All experiments were repeated independently at least three biological replicates. Scale bar = 50 μm. *< 0.05, **< 0.01, ***< 0.001, compared with the control group. ^#^< 0.05, ^##^< 0.01, ^###^< 0.001, compared with the IL-1β group

To investigate if the BMSC-derived exosomes have an effect on cartilage matrix function, the expressions of extracellular proteolytic enzymes in cartilage degradation (catabolic markers, MMP13 and ADAMTS5) and anabolic markers (COL2A1, ACAN) were assessed in IL-1β-treated chondrocytes [35]. As shown in Fig. 2a, IL-1β treatment significantly downregulated COL2A1 and ACAN and upregulated MMP13 and ADAMTS5 mRNA expressions (all *P* < 0.01); however, exosome pre-treatment (40 μg) significantly attenuated IL-1β-induced changes in the expression of these genes (all *P* < 0.05).

To confirm the expression of collagen type II protein (COL2A1), the specific marker in both hyaline and articular cartilage, BMSCs were induced to chondrogenic differentiation and subjected to western blot. As shown in Supplementary Figure 3, a high-level expression of collagen type II protein (142 kD) could be observed in both chondrocytes (monolayer chondrocytes) and BMSCs induced to chondrogenic differentiation for 14 days (pellet culture chondrocytes). Additionally, western blot (Fig. 2b) and immunofluorescence (Fig. 2c) both demonstrated that exosome pre-treatment (40 μg) significantly attenuated IL-1β-induced downregulation of COL2A1 and upregulation of MMP13 proteins (all *P* < 0.01).

In addition, in the in vitro chondrocyte model, PCR (Supplementary Fig. 4A) and western blot assays (Supplementary Fig. 4B) showed that fibrocartilage marker COL1A1 expression was at a low level in normal chondrocytes (hyaline cartilage), but was upregulated in IL-1β-induced chondrocyte degeneration model (both *P* < 0.001), which can be downregulated by exosomes (all *P* < 0.01).

Taken together, these results suggested that BMSC-derived exosomes protected chondrocytes (hyaline cartilage) from IL-1β-induced cartilage damage. In addition, a higher dose of exosomes provided a better protective effect.

### Exosomes alleviate cartilage damage in sodium iodoacetate-induced experimental osteoarthritis rats

Next, we attempted to evaluate the protective effect of exosomes on osteoarthritis in vivo. A rat model was established by injection of sodium iodoacetate. The animals were divided into three groups: sham group, OA group, and EXO group which were injected with exosomes (40 μg/week) for 6 weeks.

**Fig. 3:**
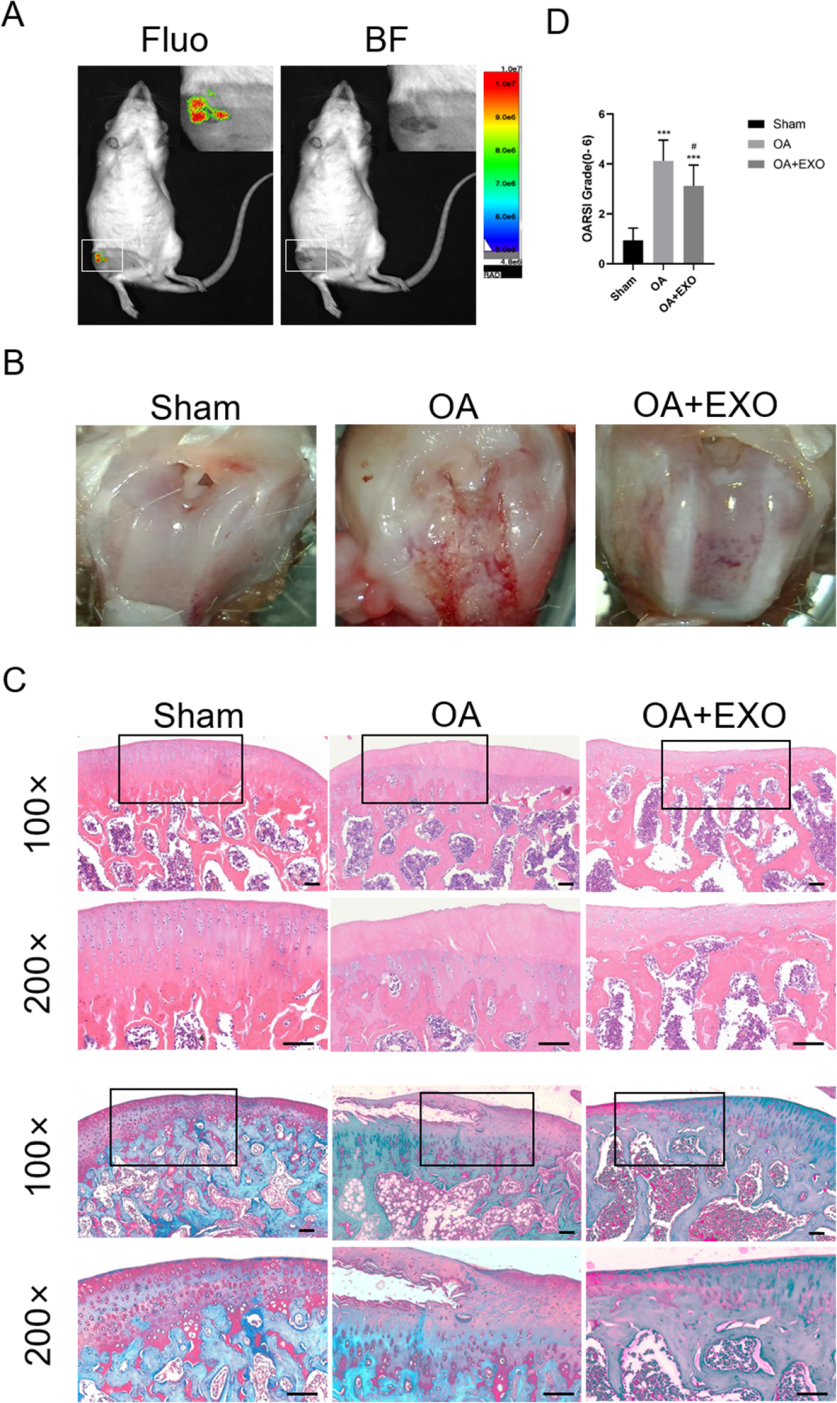
Exosomes alleviate cartilage damage in OA rats. **a** In vivo imaging of the rat after injection of the exosomes in the joint cavity. Fluo, fluorescence, BF, brightfield. **b** Gross morphological images of rat’s knee. **c** Images of H&E and Safranin O/Fast Green staining of knee joint specimens. Scale bar = 50 μm. **d** Osteoarthritis Research Society International (OARSI) score for the cartilage among different groups. ***< 0.001, compared with the sham group. ^#^< 0.05, compared with the OA group. *n* = 8 for each group

After injection of the exosomes in the joint cavity, in vivo imaging of the rat showed that exosomes accumulated in the joint cavity of the injection side, suggesting that the exosomes functioned locally in the joint cavity of the OA rat (Fig. 3a).

The gross morphological images of the rat’s knee were shown in Fig. 3b. In the OA group, the articular surface was rough and ulcerated, and osteophytes formed around the joints. While in the OA + EXO group, joint injuries were repaired to some extent.

At 6 weeks after the model was established, the knee joint specimens of animals were collected for H&E and Safranin O/Fast Green staining (Fig. 3c). The staining showed that in the sham group, the articular cartilage was clear with a smooth and intact surface. In the OA group, the surface of the articular cartilage was rough and fractured. Some part of the subchondral bone was exposed, and the synovium exhibited hyperemia and significant hyperplasia. Compared to the sham group, the cartilage layer of the OA group was lightly stained and thinner. The subchondral bone was thickened with disordered structure and formation of multiple osteophytes, suggesting the OA model was successfully established. The EXO group exhibited a small number of defects and fractures on the cartilage surface, suggesting that exosome treatment attenuated cartilage damage in OA animals.

The OARSI scores of the knee joint specimens were significantly higher in the OA group than in the sham group (4.1 ± 0.8 vs. 0.9 ± 0.5, *P* < 0.001). Exosome treatment significantly reduced the OARSI scores as compared with the OA group (3.1 ± 0.8 vs. 4.1 ± 0.8, *P* < 0.05) (Fig. 3d).

**Fig. 4:**
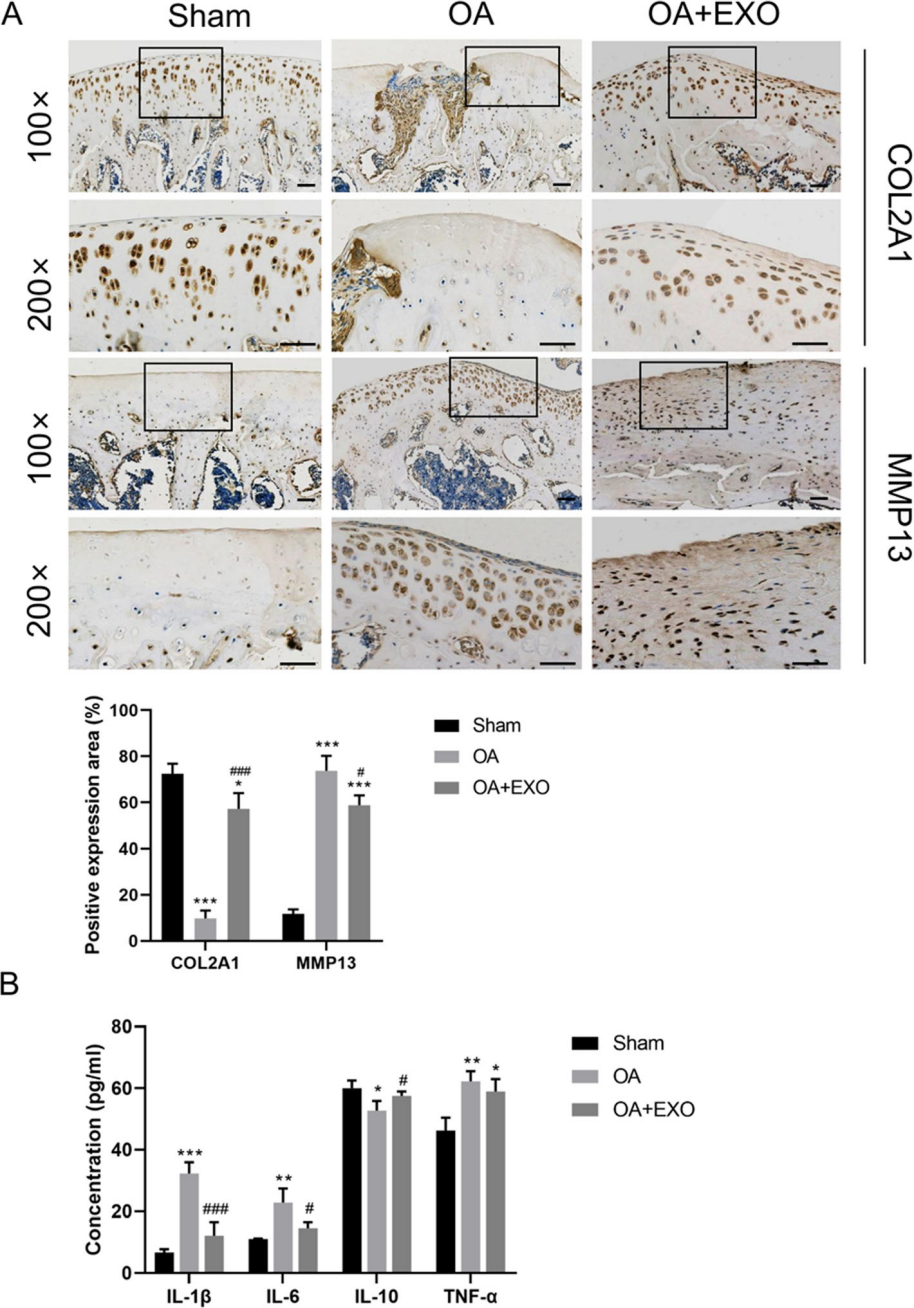
**a** Immunohistochemical staining of COL2A1 and MMP13 proteins in the cartilage tissue. Scale bar = 50 μm. **b** Detection of serum inflammatory factors in OA rats by ELISA. *< 0.05, **< 0.001, ***< 0.001, compared with the sham group. ^#^< 0.05, ^###^< 0.001, compared with the OA group. *n* = 8 for each group

To further clarify the relationship between COL2A1 and extracellular matrix (ECM), IHC staining of COL2A1 was performed in the knee cartilage layer of the in vivo knee joint OA model. The results showed that with the increase of COL2A1 in chondrocytes, the expression of COL2A1 in ECM also increased (Fig. 4a). In addition, the expression of COL2A1 in the chondrocytes and ECM was significantly higher in the OA + EXO group than in the OA group (*P* < 0.001, Fig. 4a). Meanwhile, MMP13 protein was significantly upregulated in the cartilage tissue of OA animals (*P* < 0.001, Fig. 4a), and exosome treatment can attenuate OA-induced upregulation of MMP13 protein (both *P* < 0.05, Fig. 4a).

The expression of fibrocartilage marker COL1A1 was determined in the animal model. The results showed that COL1A1 expression was at a low level in the normal knee cartilage layer (hyaline cartilage, the sham group). Partial expression of COL1A1 was seen in the superficial layer of fibrotic hyaline cartilage and hyperplastic fibrocartilage tissue in the OA group. In the OA + EXO group, COL1A1 expression was downregulated in the OA knee cartilage layer (Supplementary Fig. 4C).

To assess serum levels of inflammatory cytokines in OA animals, an ELISA assay was performed. It was found that the EXO treatment can significantly attenuate the upregulation of serum inflammatory cytokines IL-1β, IL-6, and TNF-α in the OA animals and promote the expression of the anti-inflammatory cytokine IL-10 (Fig. 4b, all *P* < 0.05).

### Exosomes relieved pain in OA rats

**Fig. 5:**
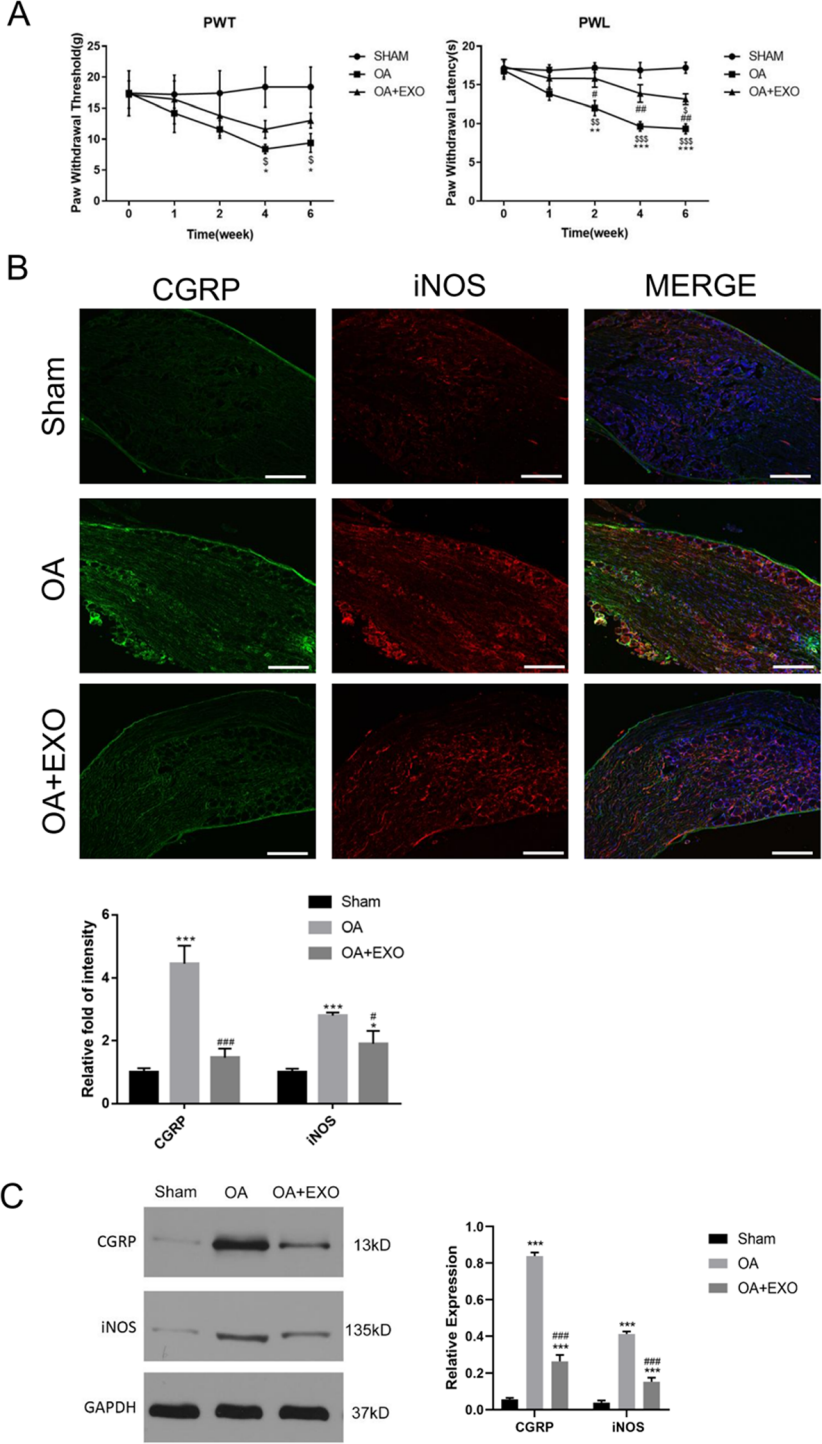
Exosomes relieved pain in OA rats. **a** PWT and PWL were used to evaluate mechanical pain sensitivity and hyperalgesia. ^$^< 0.05, ^$$^< 0.01, ^$$$^< 0.001, compared with the baseline level before model establishment (week 0). *< 0.05, **< 0.01, ***< 0.001, compared with the sham group. ^#^< 0.05, ^##^< 0.01, compared with the OA group. *n* = 8 for each group. **b** Immunofluorescence staining of CGRP and iNOS protein in the dorsal root ganglion (DRG) tissue. Scale bar = 200 μm. *< 0.05, ***< 0.001, compared with the sham group. ^#^< 0.05, ^###^< 0.001, compared with the OA group. *n* = 4 for each group. **c** Western blot analysis of protein levels of CGRP and iNOS in the DRG tissue. ***< 0.001, compared with the sham group. ^###^< 0.001, compared with the OA group. *n* = 4 for each group

To evaluate if exosome treatment can relieve pain in OA rats, PWT and PWL were used to evaluate the mechanical pain sensitivity and hyperalgesia. As shown in Fig. 5a, there was no significant difference between PWT and PWL in the sham group (*P* > 0.05). When compared with the baseline level before the model establishment (week 0), both PWT and PWL values were significantly lower at week 4 and week 6 in the OA group (all *P* < 0.05), while PWL value was lower at week 6 in the EXO group. Similarly, PWT and PWL were significantly lower at 4 and 6 weeks in the OA group as compared with the sham group (all *P* < 0.05). There was no significant difference in PWT and PWL values between 4 and 6 weeks (*P* > 0.05). At weeks 2, 4, and 6, the PWL value was significantly improved in the EXO group (all *P* < 0.05, Fig. 5a, right panel) as compared with the OA group. However, although PWT was improved in the EXO group as compared with the OA group, the difference did not reach significance (Fig. 5a, left panel).

Both immunofluorescence (Fig. 5b) and western blot (Fig. 5c) showed that the protein levels of CGRP and iNOS were significantly upregulated in DGR tissues of OA rats as compared with the sham group at 6 weeks after model establishment, suggesting that the OA rats had a combination of inflammatory and neuropathic pain. Moreover, the protein levels of CGRP and iNOS were significantly reduced in the EXO group as compared with the OA group, which indicated that BMSC-derived exosomes had a protective effect of pain relief for OA rats.

## Discussion

In this study, we investigated the effect of BMSC-derived exosomes on the cartilage damage in the rat model of osteoarthritis. In vitro study showed that exosomes can be endocytosed with chondrocytes. BMSC-derived exosome treatment significantly attenuated IL-1β-induced inhibition on the proliferation and migration of chondrocytes. Exosome treatment significantly attenuated IL-1β-induced downregulation of COL2A1 and ACAN and upregulation of MMP13 and ADAMTS chondrocytes. In the rat model of OA, exosome treatment significantly upregulated COL2A1 protein and downregulated MMP13 protein in the cartilage tissue. At weeks 2, 4, and 6, the PWL value was significantly improved in the EXO group as compared with the OA group. Moreover, exosome treatment significantly alleviated the upregulation of CGRP and iNOS in the dorsal root ganglion (DRG) tissue of the OA rat. Taken together, these results suggested that BMSC-derived exosomes can effectively promote cartilage repair and extracellular matrix synthesis, as well as alleviate knee pain in the OA rats.

Inflammation plays a vital role in the pathogenesis of OA [36], and proinflammatory cytokine IL-1β is one of the critical mediators of cartilage destruction in OA [34]. OA patients have been reported to have elevated levels of IL-1β in the synovium synovial fluid, subchondral bone, and cartilage tissue [37, 38]. In our in vitro models of OA, the inhibitory effect of IL-1β on the proliferation and migration of chondrocytes was significantly attenuated by BMSC-derived exosome treatment, suggesting a protective effect of exosomes on chondrocytes. This observation was consistent with the findings of exosomes derived from the induced pluripotent stem cell (iPSC)-derived MSCs and synovial MSCs by Zhu et al. [39] and miR-140-5p-overexpressing synovial MSCs by Tao et al. [40].

IL-1β can upregulate cartilage matrix catabolic enzymes, including MMPs and ADAMTS5, and inflammatory mediators PGE2 and NO in chondrocytes [41, 42]. MMPs are a class of proteinases responsible for the degradation of collagen-II and proteoglycans in the articular cartilage, which play vital roles in extracellular matrix degradation in OA [43, 44]. MMP13 is an important member [45]. It has been shown that MMP13 activity is elevated in human OA cartilage and experimental OA animal models [46]. ADAMTS protein family is also implicated in cartilage degradation in OA, especially the ADAMTS5 [34]. Therefore, MMP13 and ADAMTS5 are used as catabolic markers, while COL2A1 and ACAN are used as the anabolic markers for cartilage metabolism. In the current study, BMSC-derived exosomes inhibited IL-1β-induced upregulation of MMP13 and ADAMTS5 (catabolic markers) and downregulation of COL2A1 and ACAN (anabolic markers) in rat chondrocytes. In addition, the animal study also revealed that exosome treatment downregulated MMP13 and upregulated COL2A1 in the cartilage tissue of OA rats. Our results are in line with previous studies [24, 27]. Our IHC also revealed that fibrocartilage marker COL1A1 was significantly unregulated in the fibrotic hyaline cartilage surface and hyperplastic fibrocartilage tissue, and exosome treatment can alleviate COL1A1 expression in the knee cartilage of OA animals. Combined with the results of COL2A1, these observations suggested that BMSC-derived exosomes had a chondroprotective effect and promoted cartilage (hyaline cartilage) repair in OA rats.

**Fig. 6:**
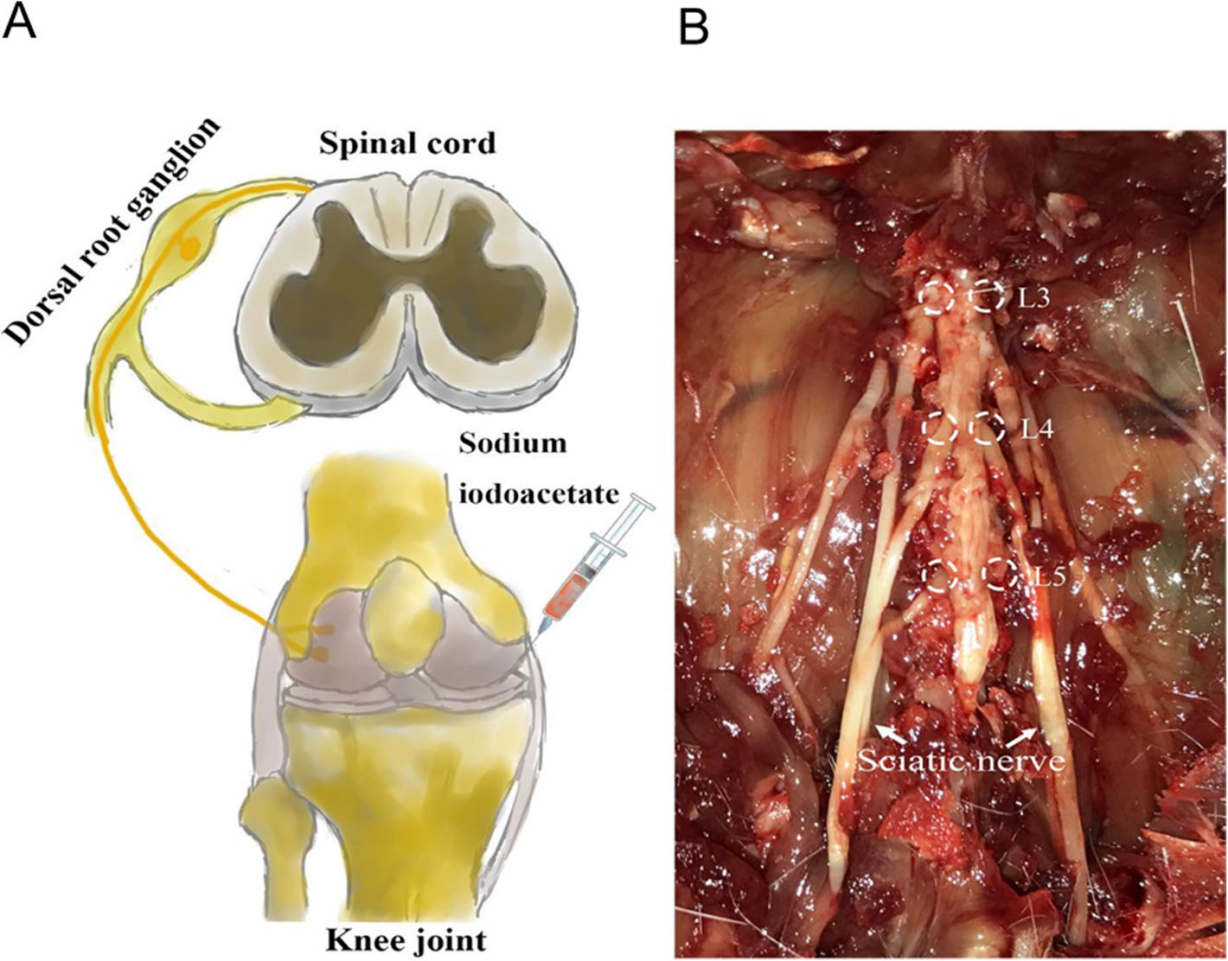
Schematic diagram of knee joint pain caused by intraarticular injection of sodium iodoacetate to establish the rat model of knee joint OA (**a**). Neuronal damage and excitability increase in L3–5 DRG are important causes of pain and peripheral sensitization, which participate in the pathogenesis of OA pain (**b**)

Although the efficacy of MSC-derived exosomes on functional recovery in the animal models of OA has been recently demonstrated [24–28], however, the therapeutic effect of BMSC-derived exosomes on pain relief has not been investigated. It has been shown that both intraarticular injection of sodium iodoacetate and partial medial meniscus resection of the knee joint OA model can induce histological changes and pain-related behavioral changes in the knee joint, but the sodium iodoacetate-induced OA model has more pain characteristics in line with clinical symptoms (Fig. 6) [32]. Our study showed that PWT and PWL were significantly lower in the OA group than in the sham group at 4 weeks after sodium iodoacetate injection, and peaked after 4 weeks, indicating that hyperalgesia and allodynia are involved in the pathogenesis of OA pain, which is consistent with Mapp et al.’s findings [47]. Hyperalgesia and allodynia are characteristic manifestations of neuropathic pain and are the result of central sensitization and peripheral sensitization of neuropathic pain. Our pain assessment demonstrated that at weeks 2, 4, and 6, the PWL value was significantly higher in the OA + EXO group than in the OA group, suggesting that exosome treatment can relieve OA pain in the rat model of OA. Chronic pain can be classified into inflammatory pain and neuropathic pain [48]. Inflammatory pain is generated from continuous stimulation to nociceptors by chronic inflammation [49], while neuropathic pain results from damage or dysfunction to the nervous system [50]. Knee OA is generally classified as nociceptive (inflammatory) pain; however, evidence suggests that the neuropathic component is complicated in some cases of OA pain [51]. CGRP is implicated in the central sensitization of neuropathic pain, CGRP is generally involved in the transmission of nociceptive information and pain sensitization in the peripheral and spinal cords, and DRG neuronal injury is an important cause of neuropathic pain and pain sensitization. CGRP is generally involved in the transmission of nociception and pain sensitization in the peripheral nerves and spinal cord, and DRG neuronal injury is an important cause of neuropathic pain and pain sensitization [52]. iNOS is an inflammatory marker. Under the stimuli of proinflammatory factors, iNOS is rapidly expressed and produces NOs, which induces an inflammatory cascade and promotes the development of inflammation. In this study, the protein levels of CGRP and iNOS were significantly upregulated in DRG tissue of OA rats as compared with the sham group at 6 weeks after model establishment, suggesting that the OA rats had neuronal damage and increased inflammatory response in DRG tissue, resulting in a combination of inflammatory and neuropathic pain (Fig. 6). Moreover, the protein levels of CGRP and iNOS were significantly reduced in the EXO group as compared with the OA group, indicating that exosome treatment can simultaneously relieve the inflammatory and neuropathic pain in OA rats. To our best knowledge, this is the first study to demonstrate the pain-relieving effect of BMSC-derived exosomes in OA animals.

There are still some limitations to this study. First, although we observed exosome treatment effectively alleviated articular cartilage injury and pain in OA rats, we did not further investigate the molecular mechanism underlying the upstream signaling molecule. In addition, the therapeutic effect of BMSC-derived exosomes remains to be validated in a clinical trial. All these limitations should be addressed in the future study.

## Conclusions

In summary, our results demonstrated that BMSC-derived exosomes can effectively promote cartilage repair and extracellular matrix synthesis, as well as alleviate knee pain in the OA rats, which have the potential to be developed as a treatment option for the OA patients.

## Supplementary information

**Additional file 1: Supplementary Fig. 1.** Characterization of BMSCs and BMSC-derived exosomes. (A) The surface markers of BMSCs were assessed by flow cytometry. (B) The multilineage differentiation potential of BMSCs was demonstrated by Alizarin Red staining, Oil Red O staining, Alcian Blue staining, and Safranin O staining, Scale bar=200 μm. (C) The size of BMSC-derived exosomes was determined by dynamic light-scattering measurement. (D) Electron microscope image of BMSC-derived exosomes. (E) Western blot analysis indicated that these vesicles displayed exosomal surface markers, including Flotillin 1, TSG101 and CD63 and were negative for the non-exosomal marker (Calnexin).

**Additional file 2: Supplementary Fig. 2.** (A) The endocytosis of exosomes by chondrocytes was detected at different time points (6, 12, 24 h). (B) The intensity of PKH26 was quantitated and presented in a bar chart. (C) BMSCs were stained with PKH26 and compared with those with exosomal endocytosis to further demonstrate the morphological characteristic of exosomal components in chondrocytes. Scale bar=50 μm, ***<0.001, compared with the 6 h, ###<0.001, compared with the 12 h.

**Additional file 3: Supplementary Fig. 3**. Western Blot for Collagen type II protein (COL2A1). A high level expression could be observed in both chondrocytes (monolayer chondrocytes) and BMSCs induced to chondrogenic differentiation (pellet culture chondrocytes). BMSCs-exosomes pre-treatment attenuated IL1β-induced downregulation of COL2A1 in monolayer chondrocytes.

**Additional file 4: Supplementary Fig. 4.** In the in vitro chondrocyte model, PCR (A) and western blot assay (B) were performed to determine the COL1A1 expression, **<0.01, ***<0.001, compared with the control group. ##<0.01, ###<0.001, compared with the IL-1β group. (C) IHC staining of COL1A1 protein in the knee cartilage layer of the in vivo knee joint OA model. Scale bar=50 μm, ***<0.001, compared with the sham group. #, <0.05, compared with the OA group.

## Data Availability

All the data and materials were presented in the main paper.

## Abbreviations

OA: Osteoarthritis
MSCs: Mesenchymal stem cells
BMSCs: Bone marrow mesenchymal stem cells
OA: Osteoarthritis
DMEM: Dulbecco’s modified Eagle’s medium
FBS: Fetal bovine serum
H&E: Hematoxylin and eosin
OARSI: Osteoarthritis Research Society International
IHC: Immunohistochemistry
PWT: Paw withdrawal threshold
PWL: Paw withdrawal latency
SD: Standard deviation
ANOVA: Analysis of variance
DRG: Dorsal root ganglion
iPSC: Induced pluripotent stem cell
