# Supplementary figures and images for: Bone marrow mesenchymal stem cell-derived exosomes protect cartilage damage and relieve knee osteoarthritis pain in a rat model of osteoarthritis
Source: Stem Cell Res Ther. 2020 Jul 10;11:276. doi: 10.1186/s13287-020-01781-w (PMC7350730; doi:10.1186/s13287-020-01781-w)

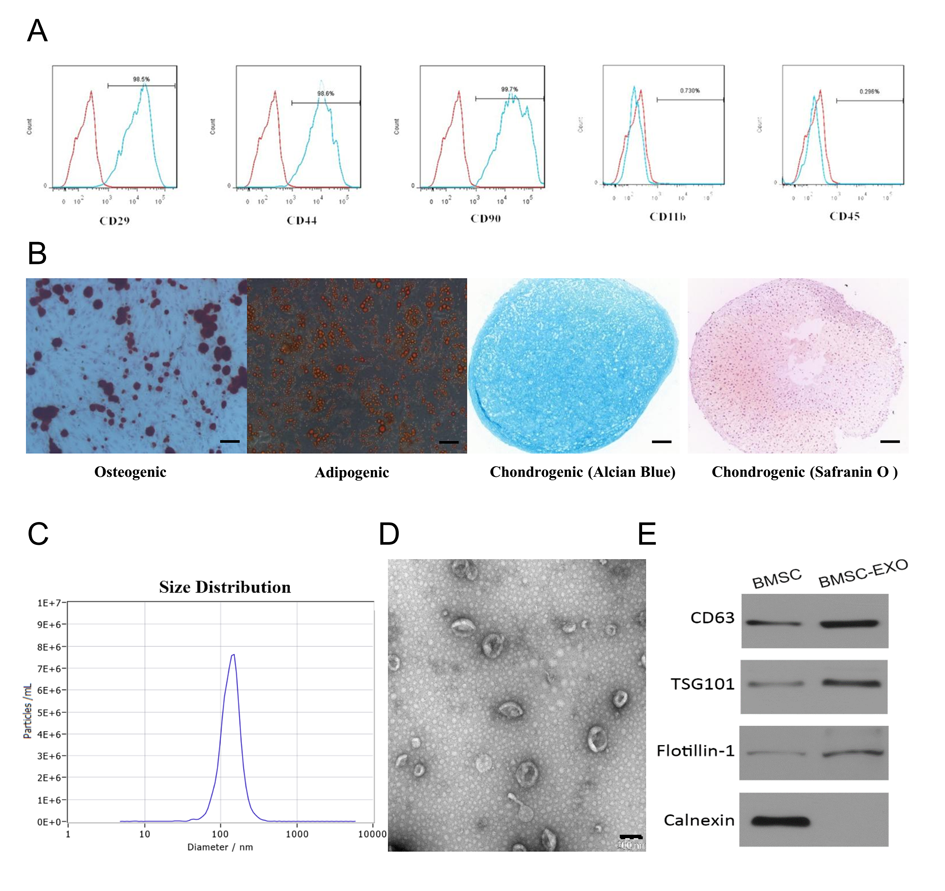

Supplement: Supplementary file 1 — Additional file 1: Supplementary Fig. 1. Characterization of BMSCs and BMSC-derived exosomes. (A) The surface markers of BMSCs were assessed by flow cytometry. (B) The multilineage differentiation potential of BMSCs was demonstrated by Alizarin Red staining, Oil Red O staining, Alcian Blue staining, and Safranin O staining, Scale bar=200 μm. (C) The size of BMSC-derived exosomes was determined by dynamic light-scattering measurement. (D) Electron microscope image of BMSC-derived exosomes. (E) Western blot analysis indicated that these vesicles displayed exosomal surface markers, including Flotillin 1, TSG101 and CD63 and were negative for the non-exosomal marker (Calnexin). [file 13287_2020_1781_MOESM1_ESM.tif]

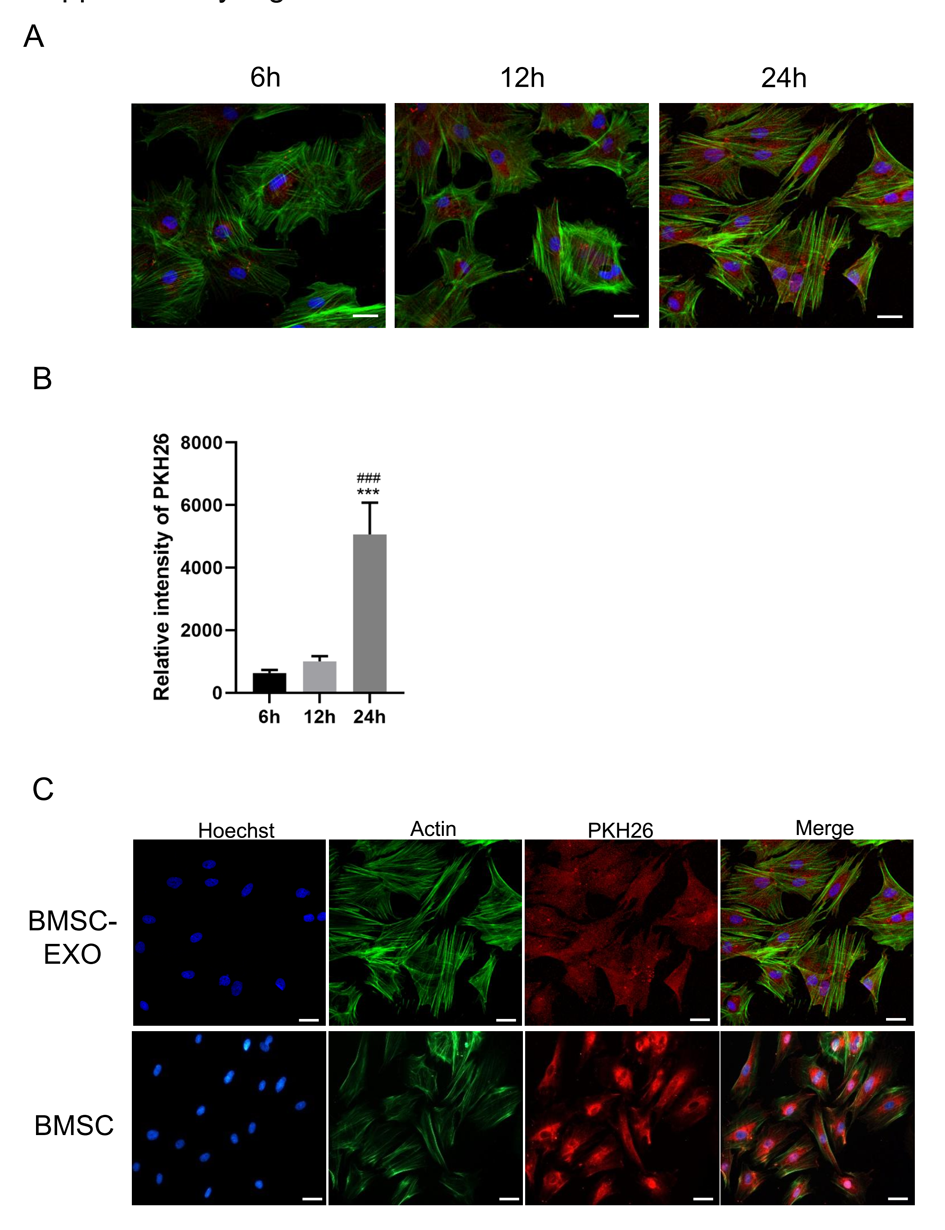

Supplement: Supplementary file 2 — Additional file 2: Supplementary Fig. 2. (A) The endocytosis of exosomes by chondrocytes was detected at different time points (6, 12, 24 h). (B) The intensity of PKH26 was quantitated and presented in a bar chart. (C) BMSCs were stained with PKH26 and compared with those with exosomal endocytosis to further demonstrate the morphological characteristic of exosomal components in chondrocytes. Scale bar=50 μm, ***<0.001, compared with the 6 h, ###<0.001, compared with the 12 h. [file 13287_2020_1781_MOESM2_ESM.tif]

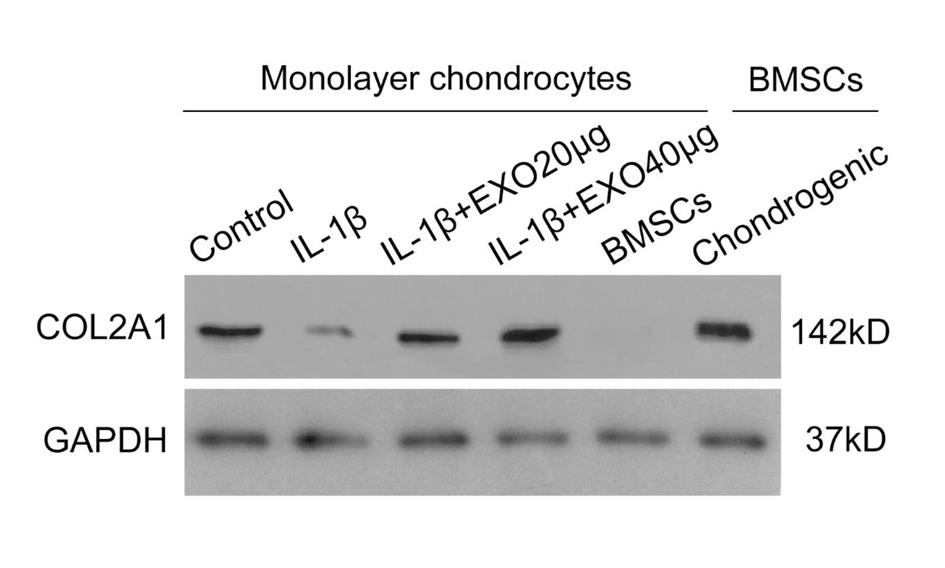

Supplement: Supplementary file 3 — Additional file 3: Supplementary Fig. 3. Western Blot for Collagen type II protein (COL2A1). A high level expression could be observed in both chondrocytes (monolayer chondrocytes) and BMSCs induced to chondrogenic differentiation (pellet culture chondrocytes). BMSCs-exosomes pre-treatment attenuated IL1β-induced downregulation of COL2A1 in monolayer chondrocytes. [file 13287_2020_1781_MOESM3_ESM.tif]

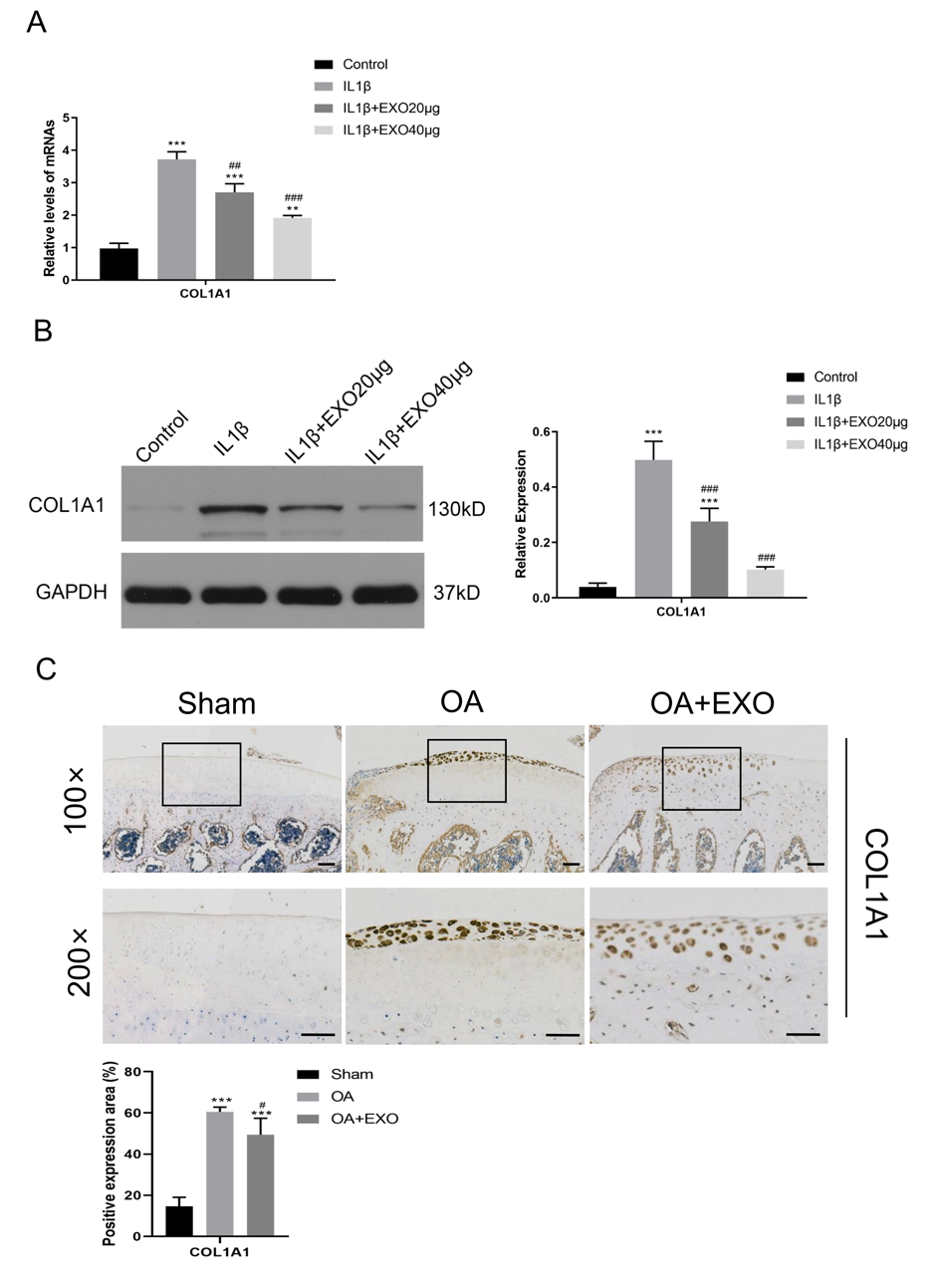

Supplement: Supplementary file 4 — Additional file 4: Supplementary Fig. 4. In the in vitro chondrocyte model, PCR (A) and western blot assay (B) were performed to determine the COL1A1 expression, **<0.01, ***<0.001, compared with the control group. ##<0.01, ###<0.001, compared with the IL-1β group. (C) IHC staining of COL1A1 protein in the knee cartilage layer of the in vivo knee joint OA model. Scale bar=50 μm, ***<0.001, compared with the sham group. #, <0.05, compared with the OA group. [file 13287_2020_1781_MOESM4_ESM.tif]
